# Validation of the Japanese Version of the Questionnaire for Impulsive-Compulsive Disorders in Parkinson's Disease-Rating Scale (QUIP-RS)

**DOI:** 10.1155/2022/1503167

**Published:** 2022-03-23

**Authors:** Maiko Takahashi, Jinsoo Koh, Shoko Yorozu, Yoshinori Kajimoto, Yoshiaki Nakayama, Mayumi Sakata, Masaaki Yasui, Yasuhiro Hiwatani, Daniel Weintraub, Hidefumi Ito

**Affiliations:** ^1^Department of Neurology, Wakayama Medical University, 811-1 Kimiidera, Wakayama, Wakayama 641-8510, Japan; ^2^Department of Neurology, Wakayama Rosai Hospital, Wakayama 640-8405, Japan; ^3^Department of Psychiatry, Perelman School of Medicine at the University of Pennsylvania, Philadelphia 19104, PA, USA

## Abstract

**Introduction:**

The Questionnaire for Impulsive-Compulsive Disorders in Parkinson's Disease (PD)-Rating Scale (QUIP-RS) was developed to assess the severity of impulsive and compulsive behaviors (ICBs) in PD. We aimed to validate the Japanese version of QUIP-RS and determine the characteristics of ICBs in Japan.

**Methods:**

We translated the QUIP-RS into Japanese, back-translated it to English, and obtained confirmation from the original author that the questionnaire remained appropriate. The participants for the validation study were 161 PD patients, identified by continuous sampling at two institutions, who were diagnosed with ICBs through a semistructured interview and completed the QUIP-RS-J. Sensitivity, specificity, and cutoff values were calculated using receiver operating characteristic (ROC) curves. Interinstitutional reliability and test-retest reliability were also assessed for a subset of participants.

**Results:**

Twenty-six (16.1%) participants were diagnosed with ICB. The optimal cutoff value of the QUIP-RS-J total score was 6, with area under the curve (AUC) = 0.889 and sensitivity/specificity of 0.92/0.71. Each subscale also showed high AUC (0.89–1.00), sensitivity (0.92–1.00), and specificity (0.71–1.00). Compared with the English version, the optimal cutoff point for binge eating was higher and hypersexuality lower. The total score tended to be higher when described by an informant.

**Conclusion:**

The present study validated the Japanese version of QUIP-RS. Use of QUIP-RS-J enables standardized assessment of ICBs and can be used in clinical research, including international multicenter studies.

## 1. Introduction

Parkinson's disease (PD) is a common neurodegenerative disease, affecting 6.1 million people in 2016 [1]. In addition to motor symptoms such as resting tremor, rigidity, and bradykinesia, patients with PD can develop nonmotor symptoms, including constipation, anosmia, depression, anxiety, and impulse control disorders (ICDs). A large epidemiological study revealed that 13.6% of PD patients experienced at least one ICD [2], and this prevalence is higher than that in the general population. ICDs include pathological gambling, hypersexuality, excessive buying, and binge eating, and ICD-related disorders include repetitive stereotypic behavior (punding), hobbyism, and dopamine dysregulation syndrome (DDS). These impulsive and compulsive behaviors (ICBs) can be induced by dopamine replacement therapy, with other risk factors being male sex, cigarette smoking history, younger age of disease onset, history of drug or alcohol abuse, novelty-seeking personality, and higher dose or long-term use of dopamine agonists [2–4]. Although ICBs can cause unproductive, harmful, and even illegal activity [2], management strategies are still under-developed. Therefore, appropriate evaluation methods are necessary for the proper management of ICBs and future research.

Recently, the Movement Disorder Society recommended the Questionnaire for Impulsive-Compulsive Disorders in Parkinson's disease (QUIP) and QUIP-Rating Scale (QUIP-RS) as evaluation tools for ICBs [5–7]. While QUIP is used as a diagnostic screening tool for ICBs, the QUIP-RS is used as a rating scale for the severity of a range of ICBs. The utility of QUIP-RS in clinical and research practice has been reported in various studies [8, 9]. Although QUIP has been translated into Japanese [10], a Japanese version of QUIP-RS has not yet been validated. QUIP-RS has been validated in various other languages and shows high sensitivity and specificity (German [11], French [12], Korean [13], and Brazilian and Portuguese [14]). As psychobehavioral disorders such as ICBs can be influenced by cultural factors [15] or language characteristics, it is necessary to validate a Japanese version of QUIP-RS. In this study, we performed validation of the Japanese version of QUIP-RS (QUIP-RS-J) and examined the characteristics of ICBs in Japan.

## 2. Methods

### 2.1. Design

We translated the original QUIP-RS into Japanese, back-translated it to English, and obtained approval from the original author (D. W.). Participants were recruited from two facilities in Japan (WMU and WR) and sampled consecutively. Participants were diagnosed with ICBs by a semistructured interview [16] using diagnostic criteria for pathological gambling [17, 18], hypersexuality [19], excessive buying [20], binge eating [17], DDS [21], and punding based on the existence of symptoms [16]. Then, they completed the translated QUIP-RS (QUIP-RS-J). The sensitivity, specificity, and cutoff value of QUIP-RS-J for the diagnosis of ICBs were calculated using receiver operating characteristic (ROC) curves, and each subscale was evaluated. In addition, we also examined the relationship between QUIP-RS-J and clinical parameters. The interfacility reliability and test-retest reliability were also evaluated using a subset of participants.

### 2.2. Participants

A total of 159 consecutive patients with PD were recruited in the two institutes: 138 patients from WMU and 21 patients from WR. The data were collected between February 2017 and March 2020 from WMU and between March 2017 and September 2017 in WR. All patients with PD fulfilled the clinical diagnostic criteria of the United Kingdom Parkinson's Disease Society Brain Bank. We assessed the Unified Parkinson's Disease-Rating Scale (UPDRS) motor score (part 3), the Barratt impulsiveness scale 11th version (BIS-11) [22], which is the most commonly used instrument designed to assess impulsivity, and the Apathy Scale [23]. The levodopa equivalent daily dose (LEDD) of dopamine agonists was calculated as previously reported [24]. This study was conducted according to the principles of the Declaration of Helsinki and approved by the Wakayama Medical University Ethics Committee (approved number: 1926). All participants provided informed consent.

### 2.3. QUIP-RS-J

The QUIP-RS is a rating scale that assesses the severity of ICBs [7]. The QUIP-RS consists of an instruction sheet and a questionnaire sheet, which responds to each disorder on a 5-point Likert scale. The scoring range for each scale is 0–16, with a total score of 0–112 points. We translated QUIP-RS into Japanese (QUIP-RS-J), and 3 patients with PD and 5 neurologists evaluated the readability. Subsequently, a native speaker translated QUIP-RS back into English, and the primary author of QUIP-RS (D. W.) approved the consistency of the questions. The QUIP-RS-J can be answered by the patient with or without an informant. A subset of patients (*n* = 9) provided data for test-retest reliability. The interval of between the test and retest had a median of 28 (interquartile range 14.5–32.5) days. The dose of levodopa or dopamine agonist was not changed between the tests and retests.

### 2.4. Diagnosis of ICBs

We developed a semistructured diagnostic interview based on the procedure of the American and German validation [6, 11]. Three trained neurologists (M. T., J. K., and Y. K.) were blinded to the results of the QUIP-RS, diagnosed each ICB, and the participants were categorized into a positive ICB group (ICBp) and a negative ICBs group (ICBn). In order to confirm the diagnostic accuracy of the semistructured interview, two neurologists (M. T. and J. K.) independently diagnosed ICBs for 26 cases with random sampling and confirmed the interrater reliability.

### 2.5. Statistical Analysis

Patient characteristics were analysed using JMP Pro14 software. Categorical variables are presented as numerals, and continuous variables are presented as mean ± standard deviation. Two group comparisons were performed using the *t*-test. Categorical variables were compared using Fisher's exact test. The QUIP-RS score, as well as each subscale score, was validated against the diagnoses of the interview, using ROC curves and area under the curves (AUC). The cutoff scores were defined using the Youden index [25]. The test-retest reliabilities of QUIP-RS were determined via interclass correlation coefficient (ICC) [26]. To explore if data quality differed between the two centers, ROC curves were calculated separately for WMU and WR. Pearson's correlation was used to evaluate the correlation between the QUIP-RS score and clinical parameters. *P* < 0.05 was considered significant.

## 3. Results

### 3.1. Sample Characteristics

**Figure 1:**
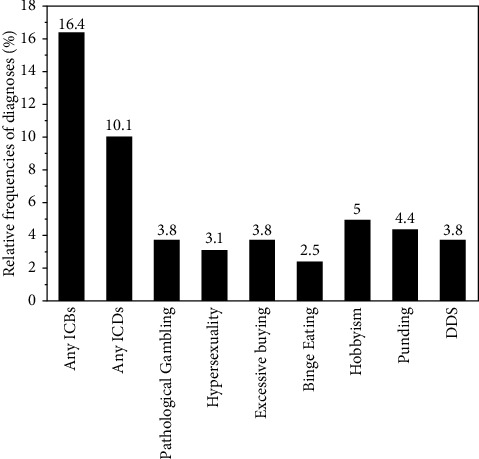
Relative frequencies of the impulsive-compulsive disorders (ICDs) by a semistructured interview. DDS, dopamine dysregulation syndrome; ICBs, impulsive-compulsive behaviors; ICDs, impulse control disorders.

The sample characteristics are given in Table 1. Participants were significantly older, milder in disease severity (UPDRS motor score), and had significantly lower doses of levodopa and dopamine agonists compared to the original United States (US) validation (age of 62.2 ± 9.6 years, UPDRS motor score of 25.7 ± 11.5, levodopa dose of 562.3 ± 493.4 mg, and dopamine agonist LEDD of 185.6 ± 171.8 mg) [7]. Twenty-six patients (16.4%) were diagnosed with one ICB, and seven patients (4.4%) were diagnosed with multiple ICBs. The frequencies of each ICB are shown in Figure 1 and tended to be less than that of the US validation.

In comparison between the ICBp and ICBn, there was no significant difference in age, gender, dopamine agonist dosages, years of education, smoking habits, and UPDRS motor scores. Conversely, ICBp was significantly younger at disease onset, had a longer disease duration, and had higher levodopa doses. The BIS-11 score tended to be higher in ICBp than in ICBn, although the difference was not statistically significant. The apathy scale was not significantly different between the two groups.

In the 26 patients evaluated for reliability between the evaluators, the diagnosis was completely consistent (22 cases without ICBs, 1 case of excessive buying, 1 case of binge eating, and 2 cases of punding).

### 3.2. QUIP-RS

**Figure 2:**
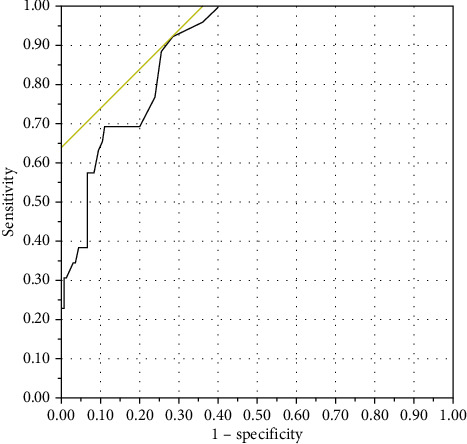
Receiver-operator characteristics (ROC) curve from the diagnosis of ICBs versus QUIP-RS score. The optimal cutoff value was 6, with a sensitivity of 0.92 and a specificity of 0.71. Area under the curve (AUC) was 0.889.

The ROC curve of QUIP-RS is shown in Figure 2. With a cutoff value of 6, the total QUIP-RS score had high AUC of 0.889, sensitivity of 0.92, and specificity of 0.71. For each subscale, the AUC, sensitivity, and specificity tended to be higher than the available original version and German validation (Table 2). Most of the cutoff values were similar, but showed different tendencies in hypersexuality and binge eating. The cutoff of binge eating score was as high as 10, while the cutoff of hypersexuality was as low as 2. Similar to the German validation, we analysed the validity of the punding and hobbyism scales as two separate scales because of high AUCs. The AUC cutoff, sensitivity, and specificity of a merge of punding and hobbyism were 0.94, 4, 1.00, and 0.81, respectively.

The QUIP-RS score was significantly higher in ICBp than in ICBn (Table 1) and was positively correlated with the BIS-11 score in a subset of participants (*n* = 127, *r* = 0.18, *p* = 0.045). Regarding sex differences, the QUIP-RS score tended to be higher in men, despite the shorter duration of illness and lower dose of dopamine agonists. In the subscale scores, hypersexuality was significantly higher in men. Forty-one patients described QUIP-RS with an informant, and their QUIP-RS score (9.7 ± 9.9) tended to be higher than those that did not use an informant (6.4 ± 10.4; *p* = 0.082).

When WMU and WR samples were compared, both samples showed high AUCs (WMU 0.89 and WR 0.99). The test-retest reliability with an ICC reliability was 0.77 for the QUIP-RS total score, although the sample size was small.

## 4. Discussion

In the present study, we validated a Japanese version of QUIP-RS, which enables the assessment of the severity of ICBs for Japanese-speaking patients in clinical practice or in clinical studies. The AUC was similar to the original version in the US and was higher than the German validation. Although the possibility of misdiagnosis through the semistructured interview cannot be completely ruled out, the diagnosis was considered highly reliable because the interrater reliability was high. The low frequency of ICBs compared to the original work was due perhaps to older age, shorter disease duration, and less severe motor symptoms in PD in participants. Furthermore, the severity of ICBs estimated by QUIP-RS was more severe with higher trait impulsivity, as previously reported [27].

All subscales showed very high sensitivities (0.92–1.0) and specificities (0.71–1.0). However, the cutoff of each subscale tended to be different when compared with the US or German version. In particular, the score for binge eating tended to be high and the score for hypersexuality tended to be low. These differences may be related to the cultural background. The obesity rate is low in Japan, and according to the 2016 survey of NCD Risk Factor Collaboration, the obesity rate with a BMI of 30 or higher is 157th for men and 199th for women among 200 countries [28]. In Japan, mild overeating tends to be considered binge eating. Therefore, Japanese participants have low shame about overeating and may be more likely report overeating in the questionnaire without concealment. In contrast, the current elderly Japanese tend to conceal sexual issues culturally and may have reported lower than the actual score. On the other hand, the higher hypersexuality observed in men was consistent with previous reports (Table 3) [2, 3, 29, 30]. Furthermore, although back translation was performed, the impressions peculiar to each language are not completely the same. As in previous reports, it should be noted that the data are not robust due to the small positive sample size of each ICB, and the individual cutoff values are of concern.

As in previous reports [31], the QUIP-RS scores tended to be higher with the cooperation of informants than with patients alone. Although this study was not designed to compare the case with and without the informants, it has been suggested that the QUIP-RS score may be underestimated when patients respond to the questionnaire with concealment or false self-assessment. Therefore, it may be desirable to provide an informant if possible.

The present data showed that ICBp had a younger age of onset, longer duration of the disease, and higher levodopa doses than ICBn, which is consistent with previous reports. Conversely, there was no significant difference in the doses of dopamine agonists. Although there have been many reports of associations between dopamine agonists and ICDs [2], a recent report has showed that ICDs are related to long-term cumulative dose of dopamine agonists [3] and may not be related to the dopamine agonist dose at the time of diagnosis with ICD. In other words, if ICD is suspected in daily practice, the dose of dopamine agonist may have already been reduced or discontinued.

Both study sites showed high AUC, so it was considered that there was no disparity in reliability between facilities. The WR sample showed an extremely high AUC due to the size of the WR sample was small (*n* = 21) and the number of ICBs was small for hobbyism (*n* = 2). As the number of cases increased, false positives and false negatives inevitably increased, and it is highly possible that the actual AUC of the WR sample was lower, similar to the WMU sample.

Other limitations include no systematic screen for cognitive function, as was performed in previous validations. However, all the participants who made the final evaluation fully understood the face-to-face questionnaire. In addition, the sample size of test-retest reliability was small. Finally, we have not evaluated whether the score changes with intervention. These issues need to be examined in future research.

## 5. Conclusions

The present study validated the Japanese version of QUIP-RS. The QUIP-RS-J showed high AUC in the diagnosis of ICBs and can assess the severity of ICBs of PD. When describing QUIP-RS-J, an informant is encouraged to be involved, if possible, as it may facilitate accurate evaluation. It should be noted that the cutoff values may differ depending on cultural background and language. The use of QUIP-RS-J enables standardized assessment of ICBs and enables clinical research, in particular, international multicenter studies.

## Figures and Tables

**Table 1:** Demographics and sample characteristics of patients with Parkinson's disease.

	Total, *n* = 159	ICBp, *n* = 26	ICBn, *n* = 133	*P* value
Age, years	69.0 ± 9.2	66.5 ± 11.5	69.5 ± 8.7	0.13^a^
Gender, f/m	92/67	15/11	77/56	1.00^b^
Age of onset, years	61.9 ± 10.2	56.3 ± 11.4	63.0 ± 9.7	0.002^a^
Duration, years	7.1 ± 5.5	10.3 ± 6.7	6.4 ± 5.1	0.001^a^
Smoking habit, *n* (%)	29 (18.2)	7 (26.9)	22 (16.5)	0.26^b^
Education, years	12.4 ± 2.3	12.5 ± 2.1	12.4 ± 2.4	0.87^a^
UPDRS motor score	15.0 ± 10.1	15.8 ± 10.4	14.8 ± 10.1	0.65^a^
DBS, *n* (%)	6 (3.8)	2 (7.7)	4 (3.0)	0.25^b^
Levodopa, mg	366.9 ± 250.0	571.2 ± 342.6	326.9 ± 206.8	<0.0001^a^
Dopamine agonist, mg/LEDD	142.5 ± 134.8	139.1 ± 117.9	143.2 ± 138.3	0.89^a^
BIS-11^∗^	59.6 ± 10.4	62.6 ± 10.2	59.1 ± 10.4	0.18^a^
Apathy scale^†^	15.2 ± 6.9	14.4 ± 7.7	15.4 ± 6.8	0.52^a^
QUIP-RS	7.2 ± 10.3	20.7 ± 14.8	4.6 ± 6.6	<0.0001^a^

Continuous variables are presented as the means ± standard deviations. ^∗^*n* = total of 127, ICBp of 19, and ICBn of 108. ^†^*n* = total of 156, ICBp of 24, ICBn of 131. ^a^*t*-test, ^b^Fisher's exact test. ICB, impulsive-compulsive behavior; UPDRS, Unified Parkinson's Disease-Rating Scale; DBS, deep brain stimulation; LEDD, levodopa equivalent daily dose; BIS-11, Barratt impulsiveness scale; QUIP-RS, Questionnaire for Impulsive-Compulsive Disorders in Parkinson's Disease-Rating Scale; ICBn, negative ICBs group; ICBp, positive ICBs group.

**Table 2:** AUC, cutoff, sensitivities, and specificities for the QUIP-RS score.

	AUC (95% CI)	Cutoff	Sensitivity	Specificity
Pathological gambling	1.00 (1.00–1.00)	5	1.00	1.00
Hypersexuality	0.97 (0.92–0.99)	2	1.00	0.92
Excessive buying	0.97 (0.92–0.99)	4	1.00	0.90
Binge eating	0.99 (0.96–1.00)	10	1.00	0.98
Hobbyism	0.94 (0.89–0.97)	4	1.00	0.83
Punding	0.96 (0.90–0.99)	1	1.00	0.84
DDS	0.97 (0.88–0.99)	2	1.00	0.85
Total ICBs	0.89 (0.82–0.93)	6	0.92	0.71

**Table 3:** Comparison between genders.

	Female *N* = 92	Male *N* = 67	*P* value^a^
Age	69.6 ± 8.9	68.2 ± 9.6	0.33
Duration	7.8 ± 6.2	6.0 ± 4.3	0.04
Age of onset	61.7 ± 10.5	62.1 ± 9.6	0.79
Levodopa, mg	376.4 ± 239.5	353.8 ± 265.0	0.57
Dopamine agonist, mg/LEDDs	162.4 ± 126.3	117.6 ± 142.4	0.04
QUIP-RS	6.21 ± 10.67	8.67 ± 9.71	0.13
Pathological gambling	0.27 ± 1.26	0.57 ± 1.77	0.22
Hypersexuality	0.13 ± 0.59	1.00 ± 1.82	<0.0001
Excessive buying	1.07 ± 2.05	1.39 ± 2.35	0.36
Binge eating	1.31 ± 2.35	2.09 ± 3.14	0.07
Hobbyism	1.47 ± 2.70	1.87 ± 2.29	0.33
Punding	0.91 ± 2.48	0.76 ± 1.95	0.68
DDS	1.04 ± 2.69	1.00 ± 2.25	0.91

Continuous variables are presented as the means ± standard deviations. ^a^*t*-test.

## Data Availability

The data used to support the findings of this study are available from the corresponding author upon request.
